# Biomarkers of Neurodegeneration and Precision Therapy in Retinal Disease

**DOI:** 10.3389/fphar.2020.601647

**Published:** 2021-01-18

**Authors:** Alessandra Micera, Bijorn Omar Balzamino, Antonio Di Zazzo, Lucia Dinice, Stefano Bonini, Marco Coassin

**Affiliations:** ^1^ Research and Development Laboratory for Biochemical, Molecular and Cellular Applications in Ophthalmological Sciences, IRCCS - Fondazione Bietti, Rome, Italy; ^2^ Ophthalmology Operative Complex Unit, University Campus Bio-Medico, Rome, Italy

**Keywords:** retinal diseases, chronic inflammation, growth factors, angiogenesis, precision medicine, neurodegeneration, pharmacological targets

## Abstract

Vision-threatening retinal diseases affect millions of people worldwide, representing an important public health issue (high social cost) for both technologically advanced and new-industrialized countries. Overall RD group comprises the retinitis pigmentosa, the age-related macular degeneration (AMD), the diabetic retinopathy (DR), and idiopathic epiretinal membrane formation. Endocrine, metabolic, and even lifestyles risk factors have been reported for these age-linked conditions that represent a “public priority” also in this COVID-19 emergency. Chronic inflammation and neurodegeneration characterize the disease evolution, with a consistent vitreoretinal interface impairment. As the vitreous chamber is significantly involved, the latest diagnostic technologies of imaging (retina) and biomarker detection (vitreous) have provided a huge input at both medical and surgical levels. Complement activation and immune cell recruitment/infiltration as well as detrimental intra/extracellular deposits occur in association with a reactive gliosis. The cell/tissue aging route shows a specific signal path and biomolecular profile characterized by the increased expression of several glial-derived mediators, including angiogenic/angiostatic, neurogenic, and stress-related factors (oxidative stress metabolites, inflammation, and even amyloid formation). The possibility to access vitreous chamber by collecting vitreous reflux during intravitreal injection or obtaining vitreous biopsy during a vitrectomy represents a step forward for an individualized therapy. As drug response and protein signature appear unique in each single patient, therapies should be individualized. This review addresses the current knowledge about biomarkers and pharmacological targets in these vitreoretinal diseases. As vitreous fluids might reflect the early stages of retinal sufferance and/or late stages of neurodegeneration, the possibility to modulate intravitreal levels of growth factors, in combination to anti-VEGF therapy, would open to a personalized therapy of retinal diseases.

## Introduction

A recent evaluation of the current prevalence and pattern of retinal diseases (RD) in industrialized and nonindustrialized countries highlights that RD are increasing worldwide representing a serious problem even under COVID-19 emergency.

RD group includes the retinitis pigmentosa, the age-related macular degeneration, the diabetic retinopathy, and the idiopathic epiretinal membrane formations. Other than genetic background, several risk factors (endocrine, metabolic, and even lifestyles influencers) have been reported for these age-linked conditions that represent a “public priority” as worldwide population is more long-lived and its life expectancy is increasing. The chronic inflammation and neurodegeneration affect the neuronal network, with different vitreoretinal interface impairment. As the vitreous chamber is significantly affected due to release of a plethora of soluble mediators, the latest diagnostic technologies of imaging (retina) and biomarker detection (vitreous) have provided a huge input at both medical and surgical levels. As per consecutive increase, the early complement activation, the immune cell recruitment/infiltration with local release of soluble mediators, and the formation of intra/extracellular deposits lead to impairment of the neuronal network, sustained by reactive gliosis. Tissue/cell aging shows a specific signal path and biomolecular profile characterized by the increased expression of several glial-derived mediators, including angiogenic/angiostatic, neurogenic, and stress-related factors (oxidative stress metabolites, inflammation, and even amyloid formation).

This review addresses the current knowledge about potential candidate biomarkers and pharmacological targets in these vitreoretinal diseases. As vitreous fluids might reflect the early stages of retinal sufferance and/or late stages of degeneration, the possibility to modulate intravitreal levels of growth factors will be also discussed as an additional way to improve treatment approaches, most of them in combination to anti-VEGF therapy.

## Precision Therapy for Retinal Diseases: State of the Art

Over the last years, numerous changes have been introduced to improve vitreoretinal surgery, such as the development of transconjunctival sutureless vitrectomy, ameliorated cutters, and new surgical approaches (Fujii et al., 2002). The possibility to collect vitreous biopsies represents a great opportunity of analysis for individualized medicine in retinal diseases (Oh and Oshima, 2014; Kasi et al., 2017; Coassin et al., 2020).

Globally, precision medicine approach represents a new way of thinking at prevention and treatment of multifactorial diseases, taking into consideration the individual variability in terms of genetic background, environment, and lifestyle. Although relatively new, the concept of precision medicine has represented for many years a fundamental aspect of healthcare outside ophthalmology. The possibility to predict and treat the disease depending on the specific local condition represents a crucial aspect of cancer therapy, and it is now extended to several other healthcare areas (Blix, 2014; Velez et al., 2018). In ophthalmology, precision medicine represents an effective approach for treating ocular tumors (Straatsma, 2018). In addition, this strategy is being applied successfully in the management of the inherited diseases (Ong et al., 2013). In the recent years, precision therapy has gained increasing interest for the management of retinal diseases. In particular, it has been recently proposed for intravitreal treatments in association with the biochemical analysis of vitreal reflux (Cacciamani et al., 2016). Collecting vitreous samples at the time of anti-VEGF intravitreal injection for wet AMD may be an effective method to elaborate further the precise clinical condition of the specific patient under treatment.

One aspect of precision medicine is the necessity of noninvasive indicators to drive the decision of the specialist. The contribution of computerized imaging, particularly the Optic Coherence Tomography (OCT) for examining the retinal layers in a noninvasive way, has improved drastically the posterior segment therapeutic approaches and reduced significantly healthcare costs, although a high percentage of senior population in industrialized countries is affected (Spaide et al., 2018). Diagnostic Imaging applied to retina and fundus has gained success worldwide, contributing to a better management of retinal diseases (Li et al., 2018). This computer-assisted technology has been recently associated with a considerable development of biomarkers coming from proteomic, metabolomic, and genetic basic and applied research investigation (Ristori et al., 2020).

## Retinal Diseases: The Mechanisms Behind Clinical Manifestations

**FIGURE 1:**
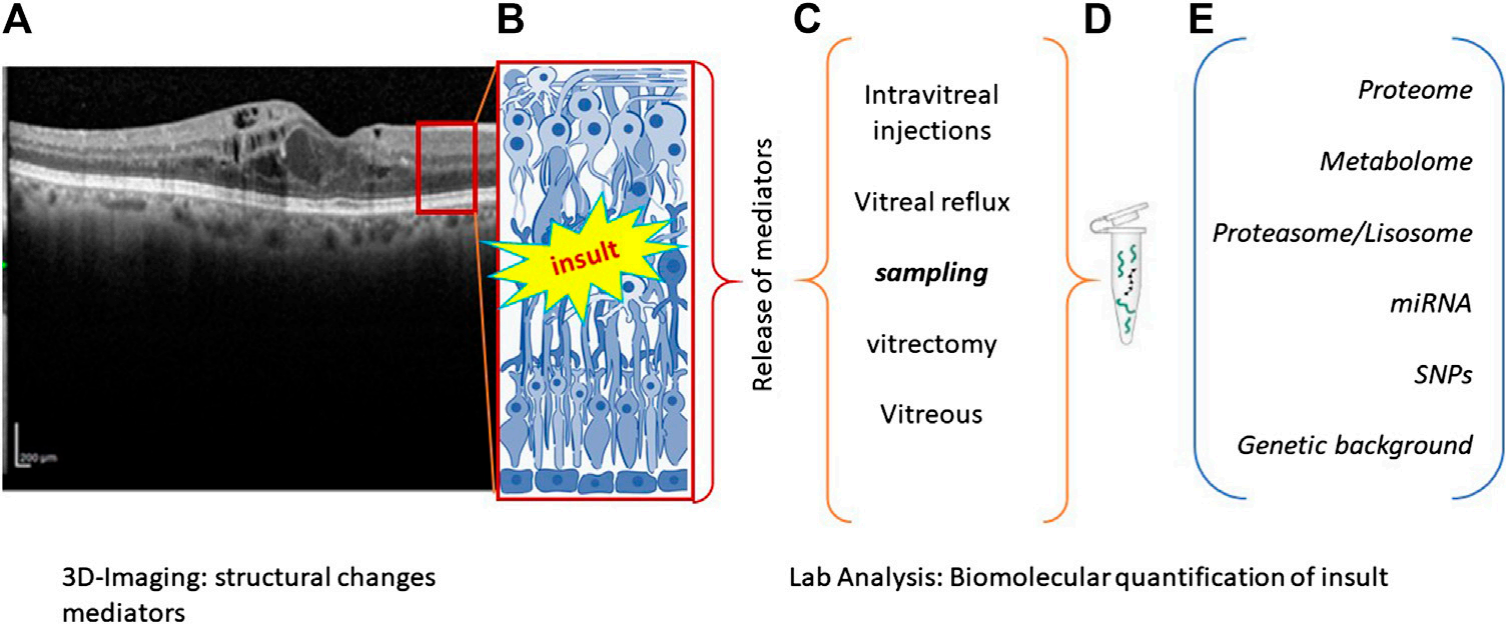
Flowchart summarizing the main steps of precision medicine. **(A)**, **(B)** Biostrumental parameters’ acquisition by computerized tomography. Representative image showing retinal layers **(A)** with a red square schematized in **(B)**. Retinal insult is followed by the release of a plethora of inflammatory mediators that can be quantified according to different sampling route **(C)**. Samples can be appropriately processed to release RNA, DNA, or proteins **(D)**, according to different new and old generation techniques **(E)**. The main biomarkers used as outcome indicator are listed in **(E)**.

Many RD have a multifactorial etiology, most probably driven by a combination of genetic and environmental factors, interacting to produce a wide range of phenotypes (Hull et al., 2014). Environmental stressors allow epigenetic modifications by influencing cellular activity and tissue response (Crick, 1970; Jafari et al., 2017). Some morphological and biostrumental biomarkers (subretinal fluid, intraretinal fluid, intraretinal cysts, hyperreflective foci, drusen/pseudodrusen, epiretinal/limiting membranes, geographic/outer retinal atrophy, and fibrovascular pigment epithelial detachment) are currently of great utility in addition to the biostrumental biomarkers provided by OCT scansion (Cacciamani et al., 2019). For sustainment, some health-recognized biomarkers are also used to discriminate between absence of, association of, or defined pathological states (Ahmed et al., 2014). The possibility to have inflammatory biomarkers, as quantified in vitreal or vitreal reflux samples or even biopsies whenever accessible, represents a step forward in grading pathological manifestation as well as in guiding the surgical decision (Figure 1). RD forms will be discussed stepwise with respect to biomarkers’ association. RD group (RP, AMD, DR, and ERMs) will be discussed below with respect to clinical manifestations and biomarker association as quantified in vitreal fluids.

### Retinitis Pigmentosa (RP)

RP is a neurodegenerative eye disease characterized by degeneration of rod/cone photoreceptors leading to night blindness, followed by a progressive mid-peripheral vision loss, and culminating in complete blindness (around mid-40s) (Hamel, 2006). RP is genetically and phenotypically heterogeneous: mutations occur between 0.025 and 0.04% of worldwide population (Van Soest et al., 1999; Dias et al., 2018). RP outcome, disease onset (age), progression rate, and secondary clinical signs are variable, even in parents and relatives (Guadagni et al., 2015). Mutant proteins implicated in some functional process of retina may trigger nonsyndromic RP, while mutations in genes working in cells/tissues result in systemic manifestations (syndromic RP) (Waters and Beales, 2011; Wheway et al., 2014; Dias et al., 2018). Mutations in proteins associated with the classical components of the phototransduction cascade (rhodopsin and phosphodiesterase), cell signaling factors (such as Crx, Nrl), disc membrane-associated glycoproteins (peripherin), ion channels (such as the light sensitive cationic ones), and the structural proteins of the cilium can contribute to disease heterogeneity (Roesch et al., 2012; Daiger et al., 2013). Genetic and environmental factors (light) are known to modulate the entire disease: faster disease progression occurring at the inferior retina may result from a modifying effect caused by light. It has been hypothesized that light sources privilege the superior visual field, implying that a greater exposure occurs at the inferior retina, accelerating whole retinal degeneration (Paskowitz et al., 2006). This aspect was particularly evident when rd10 mice were exposed to increasing light intensity: particularly, both retinal function and photoreceptors’ number decreased as a function of light-dependent exposure, with morphological alterations and loss of synaptic connectivity inside the retinal network (Kutsyr et al., 2020).

#### Biomarkers in RP

Although genetic defects in photoreceptors or retinal pigment epithelium represent the primary causes of RP development, recent studies looked at the inflammatory response as a possible contributor for RP pathogenesis (Yoshida et al., 2013). In previous studies, Yoshida and coworkers showed that: i) inflammatory cells accumulate in the vitreous cavity of RP patients, mainly younger ones; ii) RP patients with increased inflammatory cells into vitreal cavity show decreased visual function; iii) increased proinflammatory cytokines and chemokines (IL-1α, IL-1β, IL-2, IL-4, IL-6, IL-8, IL-10, IFN-γ, GRO-α, I-309, IP-10, MCP-1, MCP-2, and TARC) typify both aqueous and vitreous from RP patients (Yoshida et al., 2013). All together, these aspects suggest the presence of a “strong” chronic inflammatory reaction depending on RP pathogenesis (Ohnaka et al., 2012). Similarly, proinflammatory cytokines and chemokines were quantified in retinas of rd10 experimental model (Ikeda et al., 2002; Ohnaka et al., 2012; Semba et al., 2013; Yoshida et al., 2013; Jové et al., 2014; Zhang et al., 2015; Kohler et al., 2016). Of those factors, MCP-1 levels were significantly increased in human RP aqueous and vitreous (Ikeda et al., 2002; Nakazawa et al., 2007; Ohnaka et al., 2012; Semba et al., 2013; Yoshida et al., 2013; Jové et al., 2014; Zhang et al., 2015; Kohler et al., 2016). As known from other diseases and experimental studies, the ability of MCP-1 is to recruit monocytes, activate dendritic cells, and stimulate memory T cells at injured sites (Ikeda et al., 2002; Nakazawa et al., 2007). More interestingly, MCP-1 activates microglia, which contributes to neuronal inflammation and subsequent neuronal apoptosis (Gupta et al., 2003; Minghetti et al., 2005). Nakazawa and coworkers demonstrated that MCP-1 contributes to photoreceptor apoptosis following retinal detachment, through a microglia/macrophage activation pathway (Nakazawa et al., 2007). In human RP retinas, another study demonstrated that an activated microglia phenotype was present in the outer nuclear layer of regions populated by death committed rods (Gupta et al., 2003). These data suggest that MCP-1, in concert with other soluble mediators, may play an important role in the inflammatory reactions and degenerative process of RP (Gupta et al., 2003; Nakazawa et al., 2007).

### Age-Related Macular Degeneration (AMD)

AMD, a neurodegenerative late-onset retinal disease showing clinical and pathological aspects close to Alzheimer Diseases, is a retinal disease leading to central vision loss: it is characterized by neurodegeneration of the central region of retina and choroid (Haddad et al., 2006). Two major forms have been identified: atrophic (dry) and exudative (wet) AMD. While dry AMD (about 85% of cases) is characterized by yellow subretinal deposits (drusen) and/or retinal pigment epithelial (RPE) irregularities (hyperpigmentation/hypopigmentary changes), the wet form (around 15% of cases) is characterized by choroidal neovascularization and fibrovascular RPE detachments (Haddad et al., 2006). Both environment and genetic factors can contribute to developing and/or exacerbation of AMD (Haddad et al., 2006). The observation of 52 common and rare variants at 34 genetic loci, independently associated with late AMD, implies that genetic factors might represent a crucial aspect in the management of disease (Haddad et al., 2006). A strong genetic association in AMD pathogenesis was found for complement factor H (CFH) and complement factor I (CFI), as well as in the metalloproteinases tissue inhibitor 3 (TIMP3), due to the presence of rare coding variants (frequency < 0·1%) (Mitchell et al., 2018). Gene–environment interaction and factors such as age and race may lead to oxidative damage and inflammation (Sacca et al., 2009; Gemenetzi and Lotery, 2020). Strong association between several environmental factors and AMD has been reported and particularly the main demographic/environmental risk factors are aging, smoking, diet, fat intake, and obesity (Klein et al., 1992; Christen et al., 1996; Seddon et al., 1996; Cho et al., 2001; Seddon et al., 2003; Friedman et al., 2004). In the neovascular form of AMD, a higher risk of AMD development was associated with white populations, with respect to Hispanic and Black ones (Sommer et al., 1991; Cruickshanks et al., 1997). Protective effects came from diet (antioxidants, nuts, fish, and omega-3 fatty acids) (Age-Related Eye Disease Study Research Group, 2001). Other potential risk factors (hypertension, high cholesterol levels, and sunlight exposure) have been prospected although with no specific involvement (Seddon and Chen, 2004).

#### Biomarkers in AMD

Analytical procedures and related immunoassay for biomarker detection (screening and monitoring) would contribute significantly to the management of AMD, at both early and late disease (Nath et al., 2017). A strong biological correlation was observed between endothelial dysfunction and biomarkers of inflammation/oxidative stress. As anti-neovascular therapy is a popular strategy for AMD, the development of predictable biomarker would be of immense importance for strategizing therapeutic modalities based on the underlying pathology. Chau and coworkers determined matrix metalloproteinase- (MMP-) 2 and MMP-9 levels in the plasma collected from AMD suffering patients, highlighting that plasmatic MMP-9 levels were significantly higher in age-related maculopathy and choroidal neovascularization (CNV) groups, as compared to control groups (Chau et al., 2008). Machalinska and colleagues showed the presence of increased circulating endothelial cells (EndCs) and endothelial progenitor cells (EPCs) in AMD patients, as compared to healthy individuals. Authors highlighted that this specific increase would reflect a severe vascular disturbance (Machalińska et al., 2011). With respect to oxidative stress, Totan and coworkers showed the presence of increased Endothelin-1 (ET-1) and reduced Nitric Oxide (NO) plasmatic levels in AMD, suggesting an imbalance between vasoconstrictor and vasodilator agents, possibly reflecting either an endothelial dysfunction in AMD pathogenesis or a role of vasoconstriction in exudative AMD (Totan et al., 2015). Another study showed significant elevation of serum concentrations of IL-1α, IL-1β, IL-4, IL-5, IL-10, IL-13, and IL-17 in AMD patients compared to control subjects (Nassar et al., 2015). IL-1 is a macrophage-derived major proinflammatory cytokine acting mainly through the induction of a network of inflammatory cytokines, chemokines, and other small soluble mediators (Dinarello, 2009). IL-1 early release might elucidate, at least in part, the increased vascular exudation resulting in larger and persisting macular edema in these patients (Dinarello, 2009). Guymer et al. also demonstrated an association between elevated urinary cytokines, transforming growth factor- (TGF-) β1 and monocyte chemoattractant protein-1 (MCP-1), and AMD (Guymer et al., 2011). Therefore, the possibility to have a “urinary panel” (biomarkers) would facilitate the monitoring of disease progression or predicting vision-threatening complications or even measuring the response to treatments (Guymer et al., 2011). Some soluble receptors, taking actively part in the recognition of major proinflammatory cytokines, have gained increasing interest in the last years. For instance, the observation of increased plasma level of soluble Tumor Necrosis Factor Receptor-II (TNFR-II) in AMD supported the hypothesis of low-grade systemic inflammation in patients with AMD (Faber et al., 2015). Of crucial interest was the use of Paraoxonase-1 (PON1), an antioxidant agent used as an indicator of lipid peroxidation, to evaluate oxidative stress in patients with AMD. Authors reported a negative correlation between PON1 activity and Malondialdehyde (MDA) levels in patients with AMD. This may suggest some efficacy of antioxidant therapy to inhibit lipid peroxidation and that hypothetical agents able to increase PON1 activity could be a therapeutic option in AMD (Baskol et al., 2006).

### Diabetic Retinopathy (DR)

DR, the result of diabetes causing damage to retinal blood vessels, is an ocular disease that may have different clinical characteristics but may lead to severe visual loss (Hayreh, 2014). Disease is associated with impaired glucose metabolism and after 20 years of type-1 diabetes, 80% of patients with insulin treated type-1 diabetes and 50% of patients with type-2 diabetes will have some degree of DR (Stitt et al., 2016). According to clinical manifestation, DR is currently categorized as mild, moderate, and severe nonproliferative diabetic retinopathy (NPDR) and proliferative diabetic retinopathy (PDR) in which retinal neovascularization is present (Cehofski et al., 2017). Diet and obesity have been reported to contribute massively to the development of type 2 diabetes. By the way, heritability can account for more than 52% of the advanced PDR, showing retinal neovascularization (Seddon, 2013).

#### Biomarkers in DR

DR is a common and often severe complication of diabetes. Nevertheless, despite the wide array of treatments available for DR management, vision restoration in advanced stage of disease is difficult. Although it is well known that tight glycemic control may protect from end-organ damage, the risk of developing diabetic complications despite adequate blood sugar control may be demonstrated by validated biomarkers. Fasching et al. demonstrated that, irrespective of metabolic control, serum concentrations of intercellular adhesion molecule-1 (ICAM-1) and vascular cell adhesion molecule-1 (VCAM-1) were elevated in patients with insulin-dependent diabetes mellitus (IDDM), reflecting ongoing endothelial cell stimulation and leukocyte activation (Fasching et al., 1996). No difference in serum E-selectin concentration was detected between diabetic patients (with or without macroangiopathy) and normal subjects, suggesting the contribution of adhesion molecules in the development of atherosclerosis occurring in diabetes (Kado and Nagata, 1999). Sharma et al. reported that circulating markers of inflammation, endothelial injury, and TNF signaling were significantly associated with DR in patients with type-1 diabetes (T1Ds). TNF receptor-I (TNFR-I) and TNFR-II receptors were highly correlated, but DR are associated more strongly with TNFR-I (Sharma et al., 2015). Studies showed the importance of TNF-α system in diabetic retinal microvascular damage (Behl et al., 2008). TNF-α binding to two specific membrane receptors (TNFR-I and TNFR-II) starts a signal pathway leading to the activation of transcription factors (NF-κB and Bax) involved in the proinflammatory and apoptotic cascade (Aderka, 1996). The diabetes-related vascular complications have been correlated with C-reactive protein (CRP), TNF-α, and IL-6 in the EURODIAB Prospective Complication Study (Schram et al., 2005). Indeed, a positive correlation was reported for DR and cardiovascular inflammatory factors, highlighting that strategies focused to decrease inflammatory activity may prevent the development of vascular complications in type 1 diabetes (Schram et al., 2005). Of interest, increased plasma levels of Endothelin (ET-1), a potent endothelium-derived vasoconstrictive peptide, have been found in non-insulin-dependent diabetic (NIDDM) patients, prospecting ET-1 as new marker of vascular damage in diabetic subjects (Laurenti et al., 1997). Jacqueminet and coworkers proposed the peripheral blood MMP-9 levels as “a suitable substitute biomarker” of retinopathy in type-1 diabetes not associated with vascular complications (Jacqueminet et al., 2006). Transforming growth factor-β (TGF-β), the main inducer of extracellular matrix remodeling and associated with collagen production and fibrogenesis, was found overexpressed in NIDDM patients (Pfeiffer et al., 1996). Lee et al. showed elevated levels of circulating endothelial progenitor cells (EPCs) and serum Erythropoietin (Epo), VEGF, and Substance P (SP) which may be involved in the progression of DR, sustaining a systemic vasculogenesis rather than a local angiogenesis (Lee et al., 2006). Both level and type of serum oxidative stress products–Malondialdehyde (MDA), Conjugated Diene (CD), Advanced Oxidation Protein Products (AOPPs), protein carbonyl, and 8-hydroxydeoxyguanosine (8-OHdG)– have been reported to have a predictive role in the development and progression of DR (Pan et al., 2008).

### Idiopathic Epiretinal Membrane Formations (ERMs)

ERMs are thin and avascular sheet of fibrous tissue developing over the retinal layer, at the vitreoretinal interphase, merely at the macular area of retina, causing changes in architectonics and functioning with consequent reduced vision. ERMs etiology is idiopathic in many cases (80%) or secondary to different situations, including retinal detachment or vascular or inflammatory retinal diseases (Bottós et al., 2012). Prognostic and therapeutic decisions occur principally by OCT evaluation (Unsal et al., 2019). Vitrectomy followed by ERM peel-off is the routine surgical approach. Nevertheless, ERMs and even the associated internal limiting membrane can reform due to genetic/epigenetic influences or particular conditions (Gemenetzi and Lotery, 2020).

#### Biomarkers in ERMs

A recent study investigated the possibility to use an interplay of OCT and biochemical markers to allow a grading of ERM formation, severity, and traction entity, an indirect marker of retinal status or detach (Stevenson et al., 2016). Other than cytokine release, some additional biomarkers have been recently identified and quantified in both vitreous and tissues (Ahmad et al., 2018). Some of those inflammatory mediators were recognized as tissue remodeling actors (IL6, IL33, and IL8), implying the possibility of a direct modulation of cell migration and collagen metabolism at the vitreoretinal interface (Dinice et al., 2020). A special note should be devoted to Osteopontin, a tissue remodeling biomarker of recent attention (Dinice et al., 2020). In addition to the cell phenotyping markers (GFAP, Iba1, and CD56), other molecular targets have been observed and some oxidative ones are highlighted at both molecular and biochemical level (Marrocco et al., 2017). None of these biomarkers can be tissue or disease specific, although their increased expression as well as just their presence can be of great diagnostic and prognostic value (Strimbu and Tavel, 2010). Last, it is important to mention that some OCT parameters could be used to predict postoperative visual outcomes in patients with iERM treated with PPV (Minami et al., 2019).

## DR and Novel Potential Players for Personalized Medicine

The possibility to select some biomarkers for predictive purposes will help to define patients suitable for therapy, according to the concept of precision medicine devoted to tailored individual needs. In the last decade, RNA and protein array approaches have been implemented by the metabolomic analysis, the proteasome/lysosome analysis, and the next generation sequencing coupled to the pharmacogenomics (PGx), as additional supports for individualized therapy (Laíns et al., 2019). Metabolome and associated pathways have been tested for improving our understanding of disease pathophysiology and associated mechanisms, as recently prospected for clarifying some aspects of a cicatricial disease of the ocular surface (Laíns et al., 2019; Di Zazzo et al., 2020). All the proteome and metabolome information should be verified with conventional approaches, bypassing some limits of these multiparametric techniques (Miteva et al., 2013). As known, metabolomics is strictly dependent on the influence of external factors (external environment, nutrition habits, age, and microbiome, among others) (Patti et al., 2012). The study of metabolomic variations allows the possibility to: i) increase the understanding of disease pathophysiology at the molecular level, generating new hypotheses for disease mechanisms; ii) identify those predictive/diagnostic biomarkers; iii) assess disease progression/exacerbation; iv) elucidate the influence of environment/lifestyle exposures in disease; and finally, v) assess drug efficacy and/or toxicity as well as eventual adverse-drug reactions (Nicholson et al., 2012; Jové et al., 2014; Kohler et al., 2016).

### Epigenetic Factors

In the recent years, the epigenetic mechanisms gained attention for their promising ability to reduce the gap between environmental factors and disease development/exacerbation, by means of gene modulation (Lanza et al., 2019). Although many efforts have been carried out to elucidate genetic and environmental risk factors for retinal diseases, little is still unknown about their molecular mechanisms. Recent studies highlighted the association between epigenetic changes and incidence/progression of retinal diseases associated with visual loss (Lanza et al., 2019). While several gene mutations have been reported for RP, the mechanisms underlying photoreceptor death remain to be elucidated (He et al., 2013). A recent study carried out in an experimental model of RP (rd1 mouse) showed an increased histone deacetylase (HDAC) activity just before photoreceptor degeneration, an effect significantly reduced in the presence of HDAC inhibitors (Sancho-Pelluz et al., 2010). A reduced electroretinography response was observed in degenerated retinal cells when the retinal endoribonuclease Dicer (a helicase with rnase motif enzyme) is specifically knocked down (Damiani et al., 2008). As well, epigenetic mechanisms, including chromatin modifications, have been implicated in AMD pathogenesis (Liu et al., 2012). Merely, Suuronen and coworkers demonstrated that the addition of HDAC inhibitors resulted in expression/secretion of clusterin (a major component of drusen) by human RPE cell line, suggesting that the management of HDAC activity is important to limit or even counteract drusen formation (Suuronen et al., 2007). By using the DNA methylation microarray and bisulfite pyrosequencing applied to frozen human RPE/choroid samples (donors), Hunter and coworkers observed the hypermethylation of glutathione S-transferase isoforms mu1 and mu5 (GSTM1 and GSTM5) in AMD compared with age-matched control tissues (Hunter et al., 2012). The ability of GSTM1 and GSTM5 to reduce oxidative stress appears of great interest, as oxidative stress was hypothesized to contribute to AMD pathophysiology (Hunter et al., 2012).

In diabetic complications, the epigenetic contribution has been proposed to explain the exacerbation of retinal damage in the presence of poor glycemic control (Villeneuve and Natarajan, 2010). As observed in streptozotocin- (STZ-) treated rats (an experimental model of diabetes) with poor glycemic control, retinas and related retinal endothelial cells showed an overexpression of the histone modifiers HADC1, HADC2, and HADC8 and a reduced activity of histone H3-specific acetyltransferase (Zhong and Kowluru, 2010). Since histone-associated impairments did not reverse upon glycemia stabilization, an epigenetic-driven metabolic memory was hypothesized as major reason for DR endurance even in the case of restoration of normal circulating glucose levels (Zhong and Kowluru, 2010). Other epigenetic modifications include the expression of histone H3K4me2 associated with the transcriptional activation and decrease of superoxide dismutase gene (SOD-2), as observed in human DR retinas (donors) (Zhong and Kowluru, 2013).

### microRNAs and Pharmacogenetic Biomarkers

Several microRNAs (miRNAs) have been implicated in photoreceptor degeneration in RP, DR, and ERMs (Wang et al., 2012; Russo et al., 2017; Anasagasti et al., 2018). Briefly, miRNAs are small nucleotide non-coding RNA sequences (≈25mers) interfering negatively with gene expression, at the RNA-induced silencing complex (RIS) level, through a binding/degrading activity (nucleases associated pattern) of specific transcripts in the cytosol. Either degrading or reducing transcript activity/translation inside the ribosomal machinery, miRNAs represent key regulators for tissue development and more properly cell growth, development, and differentiation (Jeker and Marone, 2015). One miRNA can target more than one mRNA and its ability to buffer variations in gene expression due to environmental/microenvironmental changes, and not the basic cell functions, highlights their important contribution to maintaining cellular homeostasis and para-inflammation (Lee et al., 2019). Impaired or even a complete loss of miRNA activity can result in several defects and malfunctioning at both tissue and cell level (Gurtner et al., 2016). Pathological implications of miRNA dysregulation have been described for retinal tissues, under either normal or pathological states (diabetes, neovascularization) (Liu et al., 2020). The usefulness of miRNA, as diagnostic tool, has been prospected from experimental models of diabetic retinopathy. Merely, both serum and retina of diabetic mice were found to express dysregulated miRNAs implicated in the regulation of VEGF, BDNF, PPAR-α, and CREB1 expression before the retinal vasculopathy occurs (Platania et al., 2019). Recently, a strong association of specific miRNAs with the progression and severity of retinal as well as vitreoretinal impairments has been observed (Russo et al., 2017; Martins et al., 2020). Their quantification in ocular fluids has been correlated with disease staging and severity, with promising diagnostic and/or prognostic outcomes.

### Neuroprotective Factors

A unique attention has been devoted for years to the role of growth factors, including neurotrophic and angiogenic/angiostatic mediators, whose positive role is undoubted in RD. Machalinska et al. reported a marked decrease in the Pigment Epithelium-Derived Factor (PEDF) plasma levels in patients with dry AMD, whereas a significant higher level of PEDF and Vascular Endothelial Growth Factor (VEGF) was observed in the wet form, suggesting that different manifestations of AMD may be the result of altered concentrations of counterbalancing stimulators/inhibitors of angiogenesis (Machalińska et al., 2012). In the last decades, NGF has displayed interesting abilities in the visual system, working at both ocular surface and retina/optic nerve levels. The pleiotropic NGF effects have been described either *in vitro* (cell culture models) or *in vivo* (experimental models and humans), leading to the development of clinical trials devoted to demonstrating the useful NGF administration in neurodegenerative eye diseases. Other than in neurotrophic ulcer application, this neurotrophic route was particularly evident in the treatment of impaired retinal signal in experimental retinitis pigmentosa and in retinal cell death/optic nerve degeneration in response to abnormal elevated intraocular pressure (Sposato et al., 2008; Sivilia et al., 2009). Our recent observation in experimental models supports the neuroprotective effect of NGF in insulted eye, chiefly for retina and optic nerve degeneration (Rocco et al., 2017). Based on our results, NGF protective effects might be related to an increased survival of retinal ganglion cells and nerves in the optic nerve. The cell survival and neuritis outgrowth in NGF exposed were confirmed by further results, by administration of NGF/αVEGF combination implying an additional effect of the single NGF treatment (Rocco et al., 2017). With respect to NGF alone, the NGF/αVEGF combination significantly delayed and/or protected photoreceptors as well as retinal cells from degeneration. The concept of angiogenic depletion coupled to drugs and/or growth factors has been tested recently (Heier et al., 2020).

**FIGURE 2:**
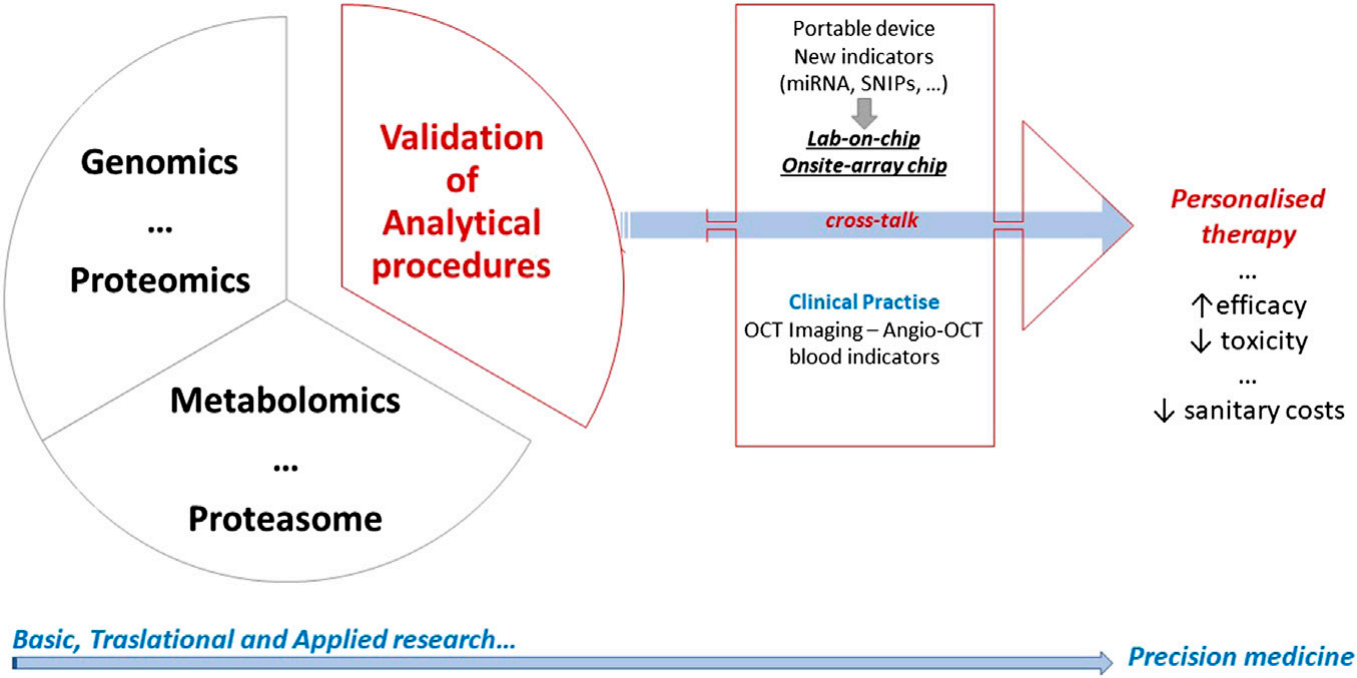
A graphical abstract summarizing the long-lasting and difficult process leading to personalized therapy. Basic and translational research crosstalk to clinical practice, and all convey to precision medicine. Overall steps in research field inform each other with the goal of improving the efficiency and effectiveness of disease prevention, diagnosis, and treatment.

All the above reported biomarkers represent a concrete support to complete the imaging information. Essentially, genomics, proteomics, metabolomics, proteasome, and pharmacogenomics can represent valid tools for precision therapy applied to retinal healthcare (Figure 2).

## Conclusion and Future Directions

As vitreous fluids might reflect the early stages of retinal sufferance and/or late stages of degeneration, the possibility to modulate intravitreal levels of growth factors, in combination to anti-VEGF therapy, would open to a new and more appropriate therapy to counteract retinal neurodegeneration. The pathways involved in this new way of thinking will be disclosed in the near future, opening to new alternative strategies of personalized therapies for a better management of retinal disorders through neuroprotection. The possibility to perform a grading of disease severity and finalize the surgical decisions is an adding value in personalized medicine.

Taken together, the above summarized findings sustain the great value of searching new research strategies for preserving the retina from neurodegeneration. The possibility to access vitreous chamber by collecting vitreous reflux during intravitreal injection or collecting vitreous biopsy at the time of vitrectomy represents a step forward for an individualized therapy. Drug response and protein signature appear to be unique in the single patient: therefore, therapies should be much as tailored as possible.

## Author Contributions

All authors have made a substantial, direct, and intellectual contribution to the work and approved it for publication.

## Funding

The study was partially supported by the Italian Ministry of Health and 5x1000 (2016) project to IRCCS-Fondazione Bietti.

## Conflict of Interest

The authors declare that the research was conducted in the absence of any commercial or financial relationships that could be construed as a potential conflict of interest.
